# Engineering natural isolates of *Saccharomyces cerevisiae* for consolidated bioprocessing of cellulosic feedstocks

**DOI:** 10.1007/s00253-023-12729-4

**Published:** 2023-09-09

**Authors:** Letitia Minnaar, Riaan den Haan

**Affiliations:** Department of Biotechnology, University of the Western Cape, Bellville, South Africa

**Keywords:** *Saccharomyces cerevisiae*, Natural isolates, Consolidated bioprocessing, Bioethanol, Cellulases

## Abstract

**Abstract:**

*Saccharomyces cerevisiae* has gained much attention as a potential host for cellulosic bioethanol production using consolidated bioprocessing (CBP) methodologies, due to its high-ethanol-producing titres, heterologous protein production capabilities, and tolerance to various industry-relevant stresses. Since the secretion levels of heterologous proteins are generally low in domesticated strains of *S. cerevisiae*, natural isolates may offer a more diverse genetic background for improved heterologous protein secretion, while also displaying greater robustness to process stresses. In this study, the potential of natural and industrial *S. cerevisiae* strains to secrete a core set of cellulases (CBH1, CBH2, EG2, and BGL1), encoded by genes integrated using CRISPR/Cas9 tools, was evaluated. High levels of heterologous protein production were associated with a reduced maximal growth rate and with slight changes in overall strain robustness, compared to the parental strains. The natural isolate derivatives YI13_BECC and YI59_BECC displayed superior secretion capacity for the heterologous cellulases at high incubation temperature and in the presence of acetic acid, respectively, compared to the reference industrial strain MH1000_BECC. These strains also exhibited multi-tolerance to several fermentation-associated and secretion stresses. Cultivation of the strains on crystalline cellulose in oxygen-limited conditions yielded ethanol concentrations in the range of 4–4.5 g/L, representing 35–40% of the theoretical maximum ethanol yield after 120 h, without the addition of exogenous enzymes. This study therefore highlights the potential of these natural isolates to be used as chassis organisms in CBP bioethanol production.

**Key points:**

*• Process-related fermentation stresses influence heterologous protein production.*

*• Transformants produced up to 4.5 g/L ethanol, ~ 40% of the theoretical yield in CBP.*

*• CRISPR/Cas9 was feasible for integrating genes in natural S. cerevisiae isolates.*

**Supplementary Information:**

The online version contains supplementary material available at 10.1007/s00253-023-12729-4.

## Introduction

The production of second-generation (2G) bioethanol from lignocellulosic biomass has gained much attention over the past two decades, due to the availability and relatively low cost of the feedstock (Claes et al. 2020; Jansen et al. 2017). Currently, the biochemical route is the preferred approach for the generation of lignocellulosic 2G bioethanol (Abo et al. 2019). However, due to the recalcitrant nature of lignocellulosic feedstocks, cellulose fibrils are inaccessible to enzymatic attack (Valenzuela-Ortega and French 2019; Zoghlami and Paës 2019), which ultimately results in low bioethanol yields and titres (Abo et al. 2019). Furthermore, subjecting lignocellulosic feedstocks to pre-treatment prior to saccharification introduces inhibitory components and/or conditions that negatively affect growth and metabolism of fermenting microorganisms (Cheah et al. 2020; Sharma et al. 2019). For these reasons, industrial applications utilise exogenous cellulase cocktails for enzymatic hydrolysis, which contribute up to 40% of the entire bioethanol production costs (Branco et al. 2019). To reduce or even eliminate the use of these enzyme cocktails, microorganisms with (1) inherent or recombinant cellulolytic activity, (2) high ethanol production yields and titres, and (3) increased robustness to various inhibitory components can be used as fermenting microorganisms, in a one-step process called consolidated bioprocessing (CBP) (Caspeta et al. 2015; Den Haan et al. 2023; Deparis et al. 2017; Valenzuela-Ortega and French 2019).

The yeast *Saccharomyces cerevisiae* is considered a promising candidate for use in 2G bioethanol production processes (Davison et al. 2016; Jansen et al. 2017), as it is widely used in various industries, and it enjoys generally regarded as safe status (Huang et al. 2014; Sheng et al. 2017; Van Wyk et al. 2018; Zhang et al. 2022). Characteristics of particular importance to industrial applications include fast growth in cheap media, high fermentation efficiency, ability to produce and tolerate high ethanol concentrations, high cell activity in acidic environments, osmo- and thermo-tolerance, and the ability to tolerate a wide variety of inhibitory conditions compared to other microorganisms (Reis et al. 2013; Xu et al. 2014). While laboratory and industrial strains of *S. cerevisiae* have defined genetic structures, the natural isolates of *S. cerevisiae* have more diverse genetic backgrounds that offers strain variants with more phenotypic variation, which is of great importance for strain improvements (Jansen et al. 2018). However, although *S. cerevisiae* possesses several important characteristics required for CBP processes, it lacks cellulolytic activity, as it does not have the inherent ability to produce the endo- and exo-glucanases required for successful enzymatic hydrolysis of cellulose (Davison et al. 2016). Several research studies have therefore aimed to genetically engineer *S. cerevisiae* strains for the heterologous secretion of the required cellulase enzymes (Den Haan et al. 2021). Various strategies that include free secretion and cell tethering of the heterologous cellulases have been reported. However, low secretion levels and increased sensitivity to fermentation stressors were also reported. Recently, Chetty et al. (2022) demonstrated improved amounts of cell-attached cellulase activities of a core set of heterologous cellulases required for 2G bioethanol production by overexpressing genes related to cellulase secretion. It was also shown that leveraging the diverse genetic backgrounds of natural strains led to improved conversion of pretreated cellulosic feedstock (Davison et al. 2020). In addition, Li and co-workers (Li et al. 2022) reported 2.2-fold increased extracellular cellulase production over the control strain, by simultaneous disruption of the native *YGP1* and overexpression of *SED5* genes. These studies show that there remains considerable potential for enhancing heterologous cellulase production through the strategic application of engineering techniques and the utilisation of various naturally occurring strain isolates.

In this study, natural isolates of *S. cerevisiae* previously reported to display industry required traits, were equipped with β-glucosidase- as well as endo- and exo-glucanase-encoding genes to evaluate their heterologous protein secretion capacity in various process relevant conditions. Superior secretors of the cellulases *Trichoderma reesei* endoglucanase II (*T.r.*EG2), *Talaromyces emersonii* cellobiohydrolase I (*T.e.*CBH1), *Chrysosporium lucknowense* cellobiohydrolase II (*C.l.*CBH2), and *Aspergillus aculeatus* $\upbeta$-glucosidase (*A.a.*BGL1) were identified by screening for individual activities in strain isolates, after successive rounds of transformation. In addition, transformed strains were screened for robustness against 2G fermentation-associated and heterologous protein secretion stresses. Finally, direct conversion of crystalline cellulose to ethanol was demonstrated in a CBP configuration. Our study demonstrates the potential role of natural strain isolates as chassis organisms for the development of industrial lignocellulose CBP processes.

## Materials and methods

### Media and culturing conditions

All chemicals and media components used were of laboratory grade and purchased from Sigma/Merck (St. Louis, MO, USA), unless otherwise stated. Microbial yeast strains were streaked from 15% (v/v) glycerol stocks stored at –80 °C onto YPD agar (1% yeast extract, 2% peptone, 2% glucose, and 2% agar) medium supplemented with 200 µg mL^−1^ geneticin G418 (Invitrogen, Waltham, MA, USA) and/or 100 µg mL^−1^ CloNAT (Werner Bioagents, Cospeda, Germany) as required, followed by incubation at 30 °C for 48–72 h (Table 1).

**Table 1:** Microbial strains used in this study

Yeast strains	Description	Reference
YI13	Natural isolate	Davison et al. (2016)
FIN1	Natural isolate	Davison et al. (2016)
YI59	Natural isolate	Davison et al. (2016)
MH1000	Natural isolate	Davison et al. (2016)
YI13_ECBE	*ENO1*_P_-*T.r.EG2*-*ENO1*_T_, *ENO1*_P_-*T.e.CBH1*-*ENO1*_T_, *ENO1*_P_-*S.f.BGL1*-*ENO1*_T_^*^	This study
FIN1_ECBE	*ENO1*_P_-*T.r.EG2*-*ENO1*_T_, *ENO1*_P_-*T.e.CBH1*-*ENO1*_T_, *ENO1*_P_-*S.f.BGL1*-*ENO1*_T_	This study
YI59_ECBE	*ENO1*_P_-*T.r.EG2*-*ENO1*_T_, *ENO1*_P_-*T.e.CBH1*-*ENO1*_T_, *ENO1*_P_-*S.f.BGL1*-*ENO1*_T_	This study
MH1000_ECBE	*ENO1*_P_-*T.r.EG2*-*ENO1*_T_, *ENO1*_P_-*T.e.CBH1*-*ENO1*_T_, *ENO1*_P_-*S.f.BGL1*-*ENO1*_T_	This study
YI13_ECBP	*ENO1*_P_-*T.r.EG2*-*ENO1*_T_, *ENO1*_P_-*T.e.CBH1*-*ENO1*_T_, *PGK1*_P_-*S.f.BGL1*-*PGK1*_T_	This study
FIN1_ECBP	*ENO1*_P_-*T.r.EG2*-*ENO1*_T_, *ENO1*_P_-*T.e.CBH1*-*ENO1*_T_, *PGK1*_P_-*S.f.BGL1*-*PGK1*_T_	This study
YI59_ECBP	*ENO1*_P_-*T.r.EG2*-*ENO1*_T_, *ENO1*_P_-*T.e.CBH1*-*ENO1*_T_, *PGK1*_P_-*S.f.BGL1*-*PGK1*_T_	This study
MH1000_ECBP	*ENO1*_P_-*T.r.EG2*-*ENO1*_T_, *ENO1*_P_-*T.e.CBH1*-*ENO1*_T_, *PGK1*_P_-*S.f.BGL1*-*PGK1*_T_	This study
YI13_BECC	*ENO1*_P_-*T.r.EG2*-*ENO1*_T_, *ENO1*_P_-*T.e.CBH1*-*ENO1*_T_, *SED1*_P_-*A.a.BGL1*-*DIT1*_T_, *PGK1*_T_-*C.l.CBH2*-*PGK1*_T_	This study
FIN1_BECC	*ENO1*_P_-*T.r.EG2*-*ENO1*_T_, *ENO1*_P_-*T.e.CBH1*-*ENO1*_T_, *SED1*_P_-*A.a.BGL1*-*DIT1*_T_, *PGK1*_T_-*C.l.CBH2*-*PGK1*_T_	This study
YI59_BECC	*ENO1*_P_-*T.r.EG2*-*ENO1*_T_, *ENO1*_P_-*T.e.CBH1*-*ENO1*_T_, *SED1*_P_-*A.a.BGL1*-*DIT1*_T_, *PGK1*_T_-*C.l.CBH2*-*PGK1*_T_	This study
MH1000_BECC	*ENO1*_P_-*T.r.EG2*-*ENO1*_T_, *ENO1*_P_-*T.e.CBH1*-*ENO1*_T_, *SED1*_P_-*A.a.BGL1*-*DIT1*_T_, *PGK1*_T_-*C.l.CBH2*-*PGK1*_T_	This study

^*^In all strains, *T.r.EG2* was targeted to chromosome 10, *BGL1* to chromosome 11, and both CBH-encoding genes to the repeated delta sequences using CRISPR-Cas9-based integration

Plasmids were propagated in *Escherichia coli* DH5 $\alpha$ by streaking strains out on Luria–Bertani (LB) agar (0.5% yeast extract, 1% tryptone, 1% NaCl, and 2% agar) supplemented with 100 µg mL^−1^ ampicillin (Roche, Basil, Switzerland), followed by overnight incubation at 37 °C (Table 2). To prepare cultures for plasmid DNA isolation, single colonies were inoculated in liquid LB media supplemented with 100 µg mL^−1^ ampicillin, followed by overnight incubation at 37 °C on a rotary wheel.

**Table 2:** Description of plasmids used in this study

Plasmids	Description	Reference
pRDH180	Contains *ENO1*_p_-*T.r.EG2*-*ENO1*_T_ gene cassette	Brevnova et al. (2011)
pMI529	Contains *ENO1*_p_-*T.e.CBH1*-*ENO1*_T_ gene cassette	Ilmén et al. (2011)
pMU-BGL1	Contains *ENO1*_p_-*S.f.BGL1*-*ENO1*_T_ gene cassette	Davison et al. (2019a, b)
ySFI	Contains *PGK1*_p_-*S.f.BGL1*-*PGK1*_T_ gene cassette	Van Rooyen et al. (2005)
pMU784	Contains *PGK1*_p_-*C.l.CBH2*-*PGK1*_T_ gene cassette	Ilmén et al. (2011)
pIBG-SSAD	Contains *SED1*_p_-*A.a.BGL1*-*DIT1*_T_ gene cassette	Inokuma et al. (2021)
pCas9-Nat	Plasmid with *cas9* expression cassette	ADDGENE
pRS42-G-ChX	gRNA scaffold plasmid that targets chromosome 10 intergenic region	Jacob et al. (2022)
pRS42-G-ChXI	gRNA scaffold plasmid that targets chromosome 11 intergenic region	Kruger and Den Haan (2022)
pRS42-G-Delta	gRNA scaffold plasmid that targets delta sequences within the yeast chromosome	Jacob et al. (2022)

### Plasmid DNA isolation, restriction digestion, and PCR amplification

Plasmid DNA isolation from *E. coli* DH5$\alpha$ cultures was performed using the cetyl trimethyl ammonium bromide (CTAB) method (Del Sal et al. 1988). To verify the sizes of each respective gene cassette and/or CRISPR gRNA sequences, isolated plasmid DNA was subjected to restriction digestion at 37 °C with *Pac*I and *Asc*I and/or *EcoR*I (Thermo Fisher Scientific, Waltham, MA, USA), respectively, followed by separation on a 1% (w/v) agarose gel. Following confirmation, gene cassettes were PCR amplified with specific primers to generate homology repair templates for use in the electro-transformation of yeast strains (Supplementary Table S1).

Purification of resolved gene cassette PCR products from agarose gels was performed using the freeze-and-squeeze method (Thuring et al. 1975), followed by phenol:chloroform:isoamyl alcohol (PCI, 25:24:1) purification. Purified DNA was then subjected to dialysis against purified water on a 0.025-µm MCE membrane filter (Merck Millipore, Burlington, MA, USA) and subsequently to quantitative spectrophotometric analysis (NanoDrop2000, Thermo Scientific) to determine the DNA concentration and purity.

### Yeast transformation and screening of putative positive transformants

Transformation of yeast strains with homology repair template DNA (*T.r.eg2, T.e.cbh1, C.l.cbh2,* and *A.a.bgl1* gene cassettes), the pCas9-NAT plasmid (Table 2), and the CRISPR plasmids (Table 2) targeting a specific intergenic region on chromosome 10 (*T.r.eg2*), chromosome 11 (*S.f.bgl1* or *A.a.bgl1*), or $\delta$-sequences (*T.e.cbh1* and *C.l.cbh2*) dispersed throughout the yeast genome were conducted as described by Cho and co-workers (Cho et al. 1999) with minor adaptations to permeabilisation of yeast cells to allow for improved transformation efficiencies (Moriguchi et al. 2016). Briefly, harvested cells were washed with sterile distilled water, followed by resuspension in lithium acetate/TE (LiOAc/TE) (0.1 M LiOAc, 10 mM Tris–HCl pH 8.0, and 1 mM ethylenediaminetetraacetic acid). Resuspended cells were then incubated at 30 °C for 45 min with shaking, prior to the incubation of cells for 15 min with 20 µL added 1 M dithiothreitol (DTT). The mixture was then centrifuged, and cells washed with sterile distilled water, followed by resuspension in electroporation buffer (1 M sorbitol, 20 mM HEPES). Competent cells were transformed with 5–10 µg homology repair template DNA and ~ 1 µg CRISPR plasmid DNA under standard electroporation conditions (1.4 kV, 200 ohms, 25 µF) using a Micropulser (Bio-Rad, Hercules, CA, USA). Following electroporation, cells were suspended in 1 mL YPD broth media supplemented with 1 M sorbitol, followed by overnight incubation at 30 °C on an orbital shaker at 180 rpm. The transformation mixture was then plated on YPD agar medium supplemented with CloNAT (100 µg mL^−1^) and/or geneticin G418 (200 µg mL^−1^) as required for 48–72 h at 30 °C.

Putative positive transformants obtained from transformation plates were then streaked on YPD media supplemented with CloNAT (100 µg mL^−1^) and/or geneticin G418 (200 µg mL^−1^), followed by incubation at 30 °C for 24–48 h, prior to inoculating overnight YPD cultures for quick yeast DNA extractions, as described by Hoffman and Winston (1987). Isolated yeast DNA was then used as DNA template to confirm the presence of integrated cellulase genes and/or integration at the correct target intergenic regions with PCR analyses, using specific primers (Supplementary Table S1). Notable strains constructed in this study were submitted to the publicly accessible Biobanks South Africa Yeast Culture Collection at the Department of Microbiology and Biochemistry, University of the Free State. Strain collection numbers are detailed in the supplementary material (Supplementary Table S2).

### Enzyme activity assays

To identify transformants with high secretory profiles for all heterologous enzymes, preliminary enzyme activity assays were conducted, by inoculating 5–10 PCR-confirmed positive transformants into 5 mL YPD liquid media for 48 h at 30 °C on an orbital shaker at 180 rpm. Superior secretors for each respective isolate were selected based on high activity profiles for all recombinant genes, with the main determinant being for CBH1 activity. Confirmed isolates were then cultivated in biological triplicates in 10 mL YPD media supplemented with 2% (w/v) glucose, under various fermentation-related conditions, namely, (1) 30 °C, (2) 37 °C, and (3) 30 °C in the presence of 3 g/L acetic acid, respectively, for 72 h with shaking at 180 rpm.

Following cultivation, EG2 activity was quantitated using the dinitrosalicylic acid (DNS) method as described by Bailey and co-workers (Bailey et al. 1992), using sodium acetate (50 mM, pH 5.0) as buffer and carboxymethyl cellulose (CMC, 1%) as substrate as recently described (Jacob et al. 2022). Briefly, cell-free supernatants and the CMC substrate were incubated at 50 °C for 60 min, followed by inhibiting the enzyme reaction with DNS. The volumetric values (U/L) obtained were normalised with the dry cell weight (DCW) of each respective isolate (Meinander et al. 1996). The enzyme activities obtained for the respective isolates were expressed as units/g DCW, where one unit (U) was equivalent to the amount of enzyme required to release 1 µmol of reducing sugar or equivalent per minute. A DNS standard curve in the range of 11–55 mM glucose was used to determine enzyme activity.

Cellobiohydrolase (CBH1) activity was quantified using soluble fluorescent 4-methyllumberiferyl-$\beta$-lactopyranoside (MULac) (Biosynth Carbosynth, Compton, UK) as substrate, as described by Ilmén and co-workers (Ilmén et al. 2011). Briefly, cell-free supernatants were incubated at 37 °C for 20 min, followed by inhibiting the enzyme reaction with sodium carbonate (1 M) prior to measuring fluorescence (excitation wavelength = 355 nm, emission wavelength = 460 nm) using a FLUOstar Omega Microplate Reader (BMG LABTECH, Ortenberg, Germany). The amount of fluorescence emitted by each sample was compared against a methylumbelliferone MU standard curve set in the range of 0.63–20 µM, and enzyme activity was expressed as units/g DCW.

For $\upbeta$-glucosidase (BGL1) activity, transformed and untransformed isolates were assayed with $\rho$-nitrophenyl-$\beta$-D-glucopyranoside (*p*NPG), as described by Van Zyl and co-workers (Van Zyl et al. 2014) with slight adaptations. Briefly, whole cell cultures were assayed at 50 °C for 30 min, prior to inhibiting the enzyme reaction with sodium carbonate (1 M). Cell cultures were then subjected to centrifugation at 3000 rpm for 2 min, after which 100 µL of cell-free supernatants were used for spectrophotometric measurements at 400 nm. Obtained volumetric values were then compared against a *p*NP standard curve in the range of 0.075–1.25 mM, and enzyme activities were expressed as units/g DCW.

### Avicel hydrolysis

To evaluate the percentage of Avicel converted by secreted cellulolytic enzymes, a substrate mixture containing 2% (w/v) Avicel PH-101 (Fisher Scientific; Hampton, NH, USA), sodium azide (0.02%), and sodium acetate (50 mM, pH 5.0) were prepared by continuous mixing to ensure homogeneity, as described by Chetty and co-workers (Chetty et al. 2022) with slight modifications. For isolates with low secreted BGL1 activity, additional commercial BGL (Novozyme-188, Sigma) was added to ensure sufficient liberation of glucose. Into a deep 96-well plate, substrate mixture and yeast culture supernatant were added at a 1:1 ratio, followed by incubation at 35 °C with shaking at 1000 rpm in a Heidolph Titramax 1000 microplate shaker/incubator. Samples were taken at 0, 24, and 48 h, to measure the amount of glucose liberated by enzymatic hydrolysis, using an adapted DNS assay procedure (Den Haan et al. 2013).

### Growth curve analyses

Cell growth determination for transformed and untransformed isolates were conducted as described by Chetty and co-workers (Chetty et al. 2022). Briefly, cell cultures were inoculated in 5 mL YPD liquid media and incubated overnight at 30 °C with shaking (180 rpm). Cultures were then inoculated to an OD_600nm_ = 0.5 in flasks containing 10 mL YPD prior to incubation in the same conditions. Samples were taken every 2 h until the stationary phase was reached. Appropriate dilutions were made at each sampling time, and OD_600nm_ readings were taken using a FLUOstar Omega Microplate Reader (BMG LABTECH, Ortenberg, Germany), applying appropriate path length correction. Growth analysis was conducted in biological triplicates, and the OD values obtained were given as the average of three repeats with their respective standard deviations.

### Strain robustness against bioethanol-related production and secretion stresses

Transformed and untransformed strain isolates were cultivated at 30 °C for 48 h in YPD liquid media, after which cell densities of cultures were measured at OD_600nm_. For standardisation, YPD was then used as diluent to obtain OD_600nm_ = 1.0 in a final volume of 1 mL. Ten-fold serial dilutions were then performed for each culture, followed by spotting 3 µL on YPD agar media supplemented with the appropriate inhibitory component. The inhibitors screened for that were specific to bioethanol production included: ethanol (8% w/v) and NaCl (1.2 M). To evaluate the response to heat stress induced by the different steps within the ethanol production process, isolates were also cultivated on YPD agar media at 30 °C and 40 °C. In addition, isolates were evaluated for their robustness against acetic acid (5 g/L) stress. To evaluate tolerance towards endoplasmic reticulum (ER) and cell wall stressors, strains were cultivated on YPD solid media supplemented with tunicamycin (1 µg mL^−1^) and Congo Red (600 µg mL^−1^), respectively. Strains were compared with regard to their sensitivity towards various inhibitors or conditions based on their growth at various dilutions.

### Fermentation of Avicel

To evaluate ethanol production of isolates on microcrystalline cellulose, isolates were inoculated in YP media supplemented with Avicel (2% w/v), as described by Chetty and co-workers (Chetty et al. 2022) with slight modifications. Briefly, isolates were pre-cultured in 50 mL YPD liquid media for 96 h at 37 °C with shaking at 180 rpm. Rubber-stoppered 20-mL glass bottles (Lasec, Cape Town, South Africa) containing 10 mL double-strength YP media (20 g/L yeast extract, 40 g/L peptone) supplemented with 40 g/L Avicel were inoculated with 10 mL pre-cultures, in biological triplicates (to achieve a final concentration of 20 g/L Avicel) followed by incubation at 30 °C for 120 h. Oxygen-limited conditions were maintained by piercing rubber stoppers with 0.8 × 25 mm syringe needles plugged with cotton wool, to act as outlets for CO_2_. Sample volumes of 1 mL were taken at 0-, 72-, and 120-h during fermentation, and subsequently stored at – 20 °C until further analysis.

Samples collected during fermentation were subjected to centrifugation at 13,000 rpm for 10 min, prior to transferring the cell-free supernatant to a clean 1.5-mL Eppendorf tube. To acidify samples, 40 µL of 10% (v/v) sulphuric acid (H_2_SO_4_) solution was added to each sample, followed by a brief vortex to mix. Samples were then filtered through a 0.22-µm filter into 2 mL HPLC (high-performance liquid chromatography) vials. Ethanol, cellobiose, acetic acid, glucose, and glycerol concentrations were determined in each sample by an HPLC equipped with a Bio-Rad guard (part # 125–0129) and refractive index (RI) detector. Compound separation was achieved on a Bio-Rad Aminex HPX-87H (part # 125–0140) 7.8 × 300 mm column at a temperature of 65 °C, with 5 mM H_2_SO_4_ as mobile phase at a flow rate of 0.7 mL/min. Values obtained for each respective compound were presented as the mean of triplicates, in g/L, with their standard deviations.

### Statistical analysis

Significant differences between enzyme activities, growth data, and/or metabolite concentrations attained were investigated using two-tailed *T*-tests, assuming unequal variance. A *p* value lower than 0.05 was deemed significant.

## Results

### Strain construction and confirmation

To create yeast strains expressing a core set of cellulases in robust strain backgrounds, we initially transformed natural and reference *S. cerevisiae* isolates with pCas9-NAT, and with each respective gRNA plasmid and homology repair cassette, corresponding to *T.r.eg2*, *S.f.bgl1*, and *T.e.cbh1*, in successive rounds of transformation. Through the CRISPR/Cas9 system, these gene cassettes could be integrated into targeted intergenic regions on Chromosome 10, -11, or in the repeated $\delta$-sequences, as previously described (Jacob et al. 2022; Kruger and Den Haan 2022). For each successive round of transformation, a homology repair cassette with its respective gRNA plasmid was transformed into the individual strain isolates (Table 2). Once successful integration of the cellulase-encoding gene was confirmed, the gRNA plasmid was cured from the strain with successive rounds of non-selective sub-culturing, prior to the next round of transformation. Successful integration of *T.r.eg2*, *T.e.cbh1*, and *S.f.bgl1* was confirmed by performing diagnostic colony PCR on putative positive transformants, which illustrated the presence of bands at 1.0 kb, 1.2 kb, and 1.0 kb, respectively (data not shown). We could therefore conclude that CRISPR-based transformation of the *S. cerevisiae* isolates was successful to allow for integration of the relevant heterologous cellulase-encoding genes.

### Enzyme activity assays

**Fig. 1:**
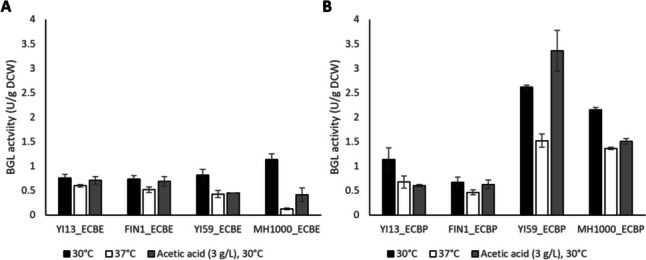
$\beta$-Glucosidase activity for recombinant natural and industrial strains of *Saccharomyces cerevisiae*. **A** *S.f.BGL1* expressed under transcriptional control of *ENO1*_*P/T*_ and **B** *S.f.BGL1* expressed under transcriptional control of *PGK1*_*P/T*_. Isolates were cultivated in three distinct growth conditions to evaluate the secretion capacity of isolates under induced stress. Results represent means of triplicate assays and standard deviation is indicated with error bars

Initial assays resulted in low BGL1 activity when *S.f.*BGL1 was expressed under transcriptional control of either the *ENO1* or *PGK1* promoter and terminator, with BGL1 activity levels ranging between 0.13 and 3.36 U/g DCW, across all conditions assayed (Fig. 1). Interestingly, differences were observed between the transformants expressing *S.f.BGL1* under transcriptional control of different regulatory sequences, despite the gene being targeted to the same chromosomal locus in the strains. While the transformed reference strains MH1000_ECBE and MH1000_ECBP displayed superior secretory capacities for the heterologous BGL1 enzyme at optimal cultivation conditions (i.e., 30 °C), the transformed natural isolates YI13_ECBE and YI59_ECBP yielded the highest specific BGL1 activity under 2G fermentation-associated conditions, namely, at higher temperature (37 °C) and/or in the presence of 3 g/L acetic acid. YI13_ECBE was noted to have superior secretory capacity for *ENO1*_*P*_-*S.f.BGL1*-*ENO1*_*T*_ at high temperature (0.59 U/g DCW) and in the presence of acetic acid (0.71 U/g DCW), which correlated to a 4.54- and 1.73-fold higher activity level than the reference strain, MH1000_ECBE, under the same conditions, respectively (Fig. 1A). Similarly, YI59_ECBP was noted to have a superior secretory capacity for *PGK1*_p_-*S.f.BGL1*-*PGK1*_T_ at high temperature (1.52 U/g DCW) and in the presence of acetic acid (3.36 U/g DCW), which correlated to a 1.12- and 2.23-fold higher specific activity to that of the reference strain, MH1000_ECBP, under the same conditions, respectively (Fig. 1B). Based on the differences observed between the transformants, and in comparison with the transformed reference strain(s), it was noted that the regulatory sequence(s) used for *S.f.*BGL1 production played a critical role in the enzyme activity level achieved by the transformants.

**Fig. 2:**
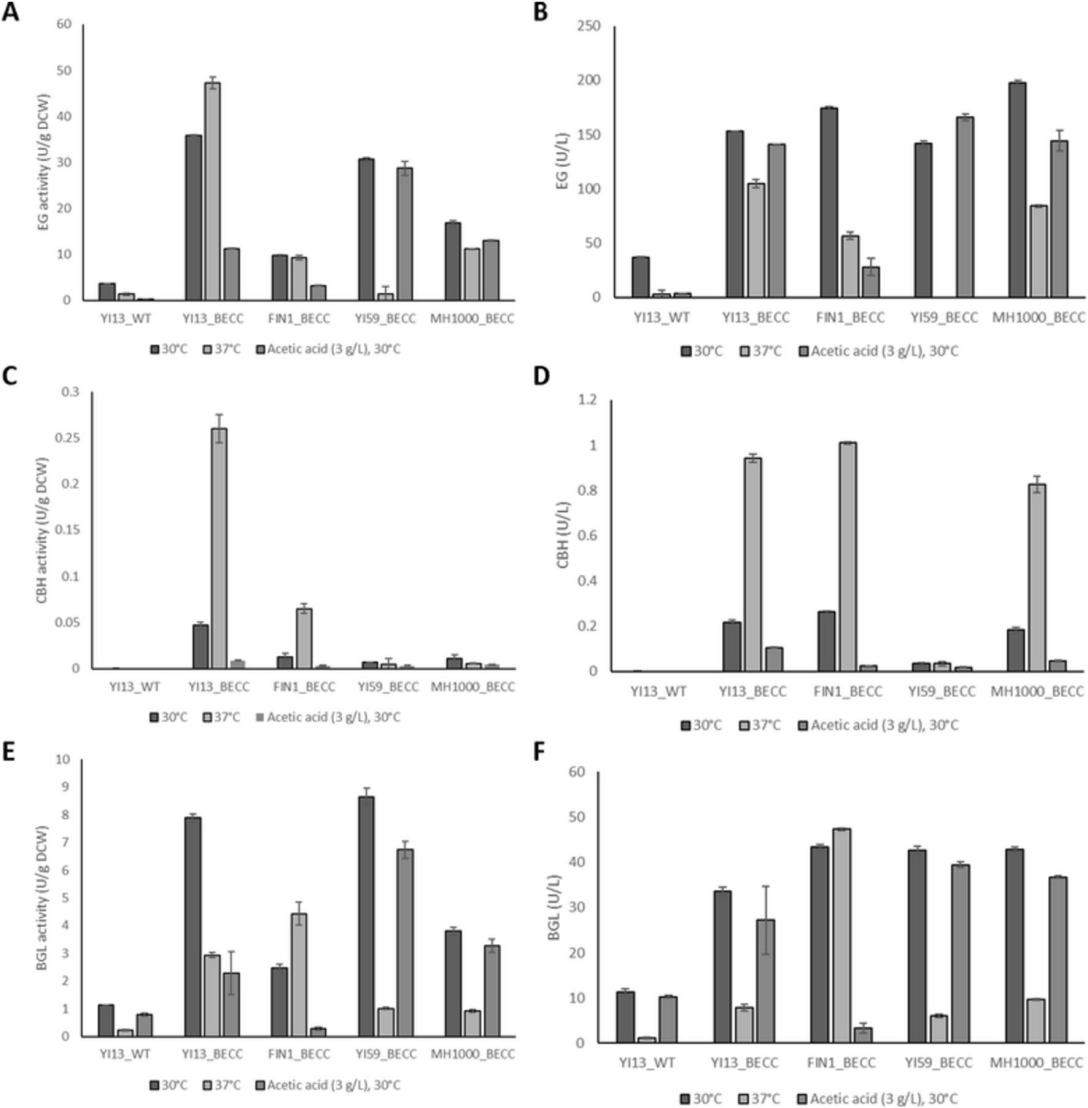
Specific (left side) and volumetric (right side) enzyme activity measurements for natural and industrial strains of *Saccharomyces cerevisiae* after 72-h incubation in the respective cultivation conditions. **A** and **B** Endoglucanase 2 (EG2) activity was measured using the dinitrosalicylic acid (DNS) method, with 1% carboxymethyl cellulose (CMC) as substrate. **C** and **D** Cellobiohydrolase 1 (CBH1) activity was measured using methylumbelliferyl-$\beta$-lactopyranoside (MUlac) as substrate. **E** and **F** β-Glucosidase 1 (BGL1) was measured using the $\rho$-nitrophenyl-$\beta$-D-glucopyranoside (pNPG) method. Results represent means of triplicate assays and standard deviation is indicated with error bars

While the use of the *PGK1*_*P/T*_ elements improved BGLI activity in most strains and conditions tested, it was still found to be comparatively low for recombinant cellulase-producing yeast strains (Chetty et al. 2022; Den Haan et al. 2021). Expression of an alternative *BGL1* was thus explored. A BGL1-encoding gene (*A.a.BGL*1) under transcriptional control of the *S. cerevisiae SED1*-derived promoter (*SED1*_*P*_) and a *S. cerevisiae DIT1*-derived terminator (*DIT1*_*T*_) sequences was used. This construct was selected based on research conducted by Inokuma and co-workers (Inokuma et al. 2014, 2016, and 2021), where the expression of a cell-surface displayed BGL1 was enhanced by optimising promoter and/or terminator sequences to allow for a 17.0-fold increase in enzyme activity compared to when conventional constitutive promoters, such as *PGK1*_*P*_ and *TDH3*_*P*_ were used. The newly constructed strains displayed significant improvements (*p* < 0.5) in BGL1 activity, with the highest activity achieved by YI59_BECC and YI13_BECC (8.65 U/g DCW and 7.89 U/g DCW) when assayed under optimal cultivation conditions (Fig. 2E). These strains were noted to yield 2.28- and 2.08-fold higher enzyme activity compared to the transformed reference strain, MH1000_BECC. However, cultivation in 2G fermentation-associated conditions yielded variable BGL1 activity levels between transformants. While YI59_BECC managed to perform relatively well under optimal cultivation conditions and in the presence of acetic acid (6.74 U/g DCW), relatively low enzyme activity was obtained at high temperature (1.01 U/g DCW). Conversely, FIN1_BECC yielded superior secretory capacity at higher cultivation temperature (4.43 U/g DCW) but yielded low enzyme activity at optimal cultivation conditions and in the presence of acetic acid (2.48 U/g DCW and 0.29 U/g DCW, respectively).

Interestingly, comparing the specific activity to that of volumetric activity between transformed natural isolates and the transformed reference strain, at optimal cultivation conditions, illustrated no significant differences between YI59_BECC, FIN1_BECC and MH1000_BECC for volumetric activity (Fig. 2F); however, up to twofold higher specific activity levels was achieved by YI59_BECC compared to FIN1_BECC and MH1000_BECC. This highlights the superior capacity of YI59_BECC to yield high BGL1 activity levels with a lower requirement in cell numbers. Further evidence substantiating this statement was shown by YI13_BECC, which displayed a lower volumetric activity than the other strains at optimal cultivation conditions but achieved relatively high specific activity levels. Thus, it was clear that YI59_BECC and YI13_BECC had superior secretory capacity for *A.a.*BGL1 compared to the other strains. Based on the differences observed between transformants, it was suggested that heterologous secretion was dependant on the hosts’ genetic background; thus, no two strains performed similarly under the same assay conditions (Davison et al. 2016). Due to the superior activity achieved with the *SED1*_*P*_*-A.a.BGL1-DIT1*_*T*_ cassette, we continued only with strains carrying this gene along with the other cellulases (i.e., *ENO1*_*P*_-*T.r.EG2*-*ENO1*_*T*_ and *ENO1*_*P*_-*T.e.CBH1*-*ENO1*_*T*_). Furthermore, these strains were also equipped with an additional cellobiohydrolase enzyme, namely, *C.l.*CBH2, which provided transformed strains with a core set of cellulases, necessary for efficient lignocellulose hydrolysis.

While YI59_BECC displayed high specific activity for *T.r.*EG2 and *A.a.*BGL1 at optimal cultivation conditions and in the presence of 3 g/L acetic acid (Fig. 2A and E), relatively low specific activity was achieved by this strain for *T.e.*CBH1 (Fig. 2C) under all conditions tested. In contrast, YI13_BECC yielded the highest specific activity for *T.r.*EG2 and *T.e.*CBHI, in agreement with Davison and co-workers (Davison et al. 2016) (Fig. 2A and C), while also displaying high specific activity for *A.a.*BGL1 (Fig. 2E), at optimal conditions and at high temperature. However, moderate to low activity was achieved for all enzymes assayed in this strain in the presence of 3 g/L acetic acid. This latter result suggested that YI13_BECC exhibited increased sensitivity to acetic acid; hence, a burden was imposed on heterologous protein production at this condition. FIN1_BECC demonstrated moderate to low activity for all the heterologous enzymes assayed, at optimal conditions and in the presence of 3 g/L acetic acid; however, moderate to high activity was achieved by this strain for CBH1 and BGL1 when cultivated at high temperature. This agreed with results obtained by Davison and co-workers (Davison et al. 2016), despite the use of episomal plasmids for expression of heterologous enzymes in that study. Based on the volumetric activity achieved for both CBH1 and EG2 for the transformed natural isolates and the reference strain, a similar pattern could be observed for the superior secretors compared to low secretors. In essence, YI59_BECC and YI13_BECC displayed similar volumetric activity levels to that of low secretors FIN1_BECC and MH1000_BECC (Fig. 2B and D), but the specific activity levels for the EG2 and CBH1 were significantly greater for YI59_BECC and YI13_BECC (Fig. 2A and C). This further illustrated that lower cell numbers were required by these latter superior secretors for high heterologous cellulase production.

### Avicel hydrolysis

**Fig. 3:**
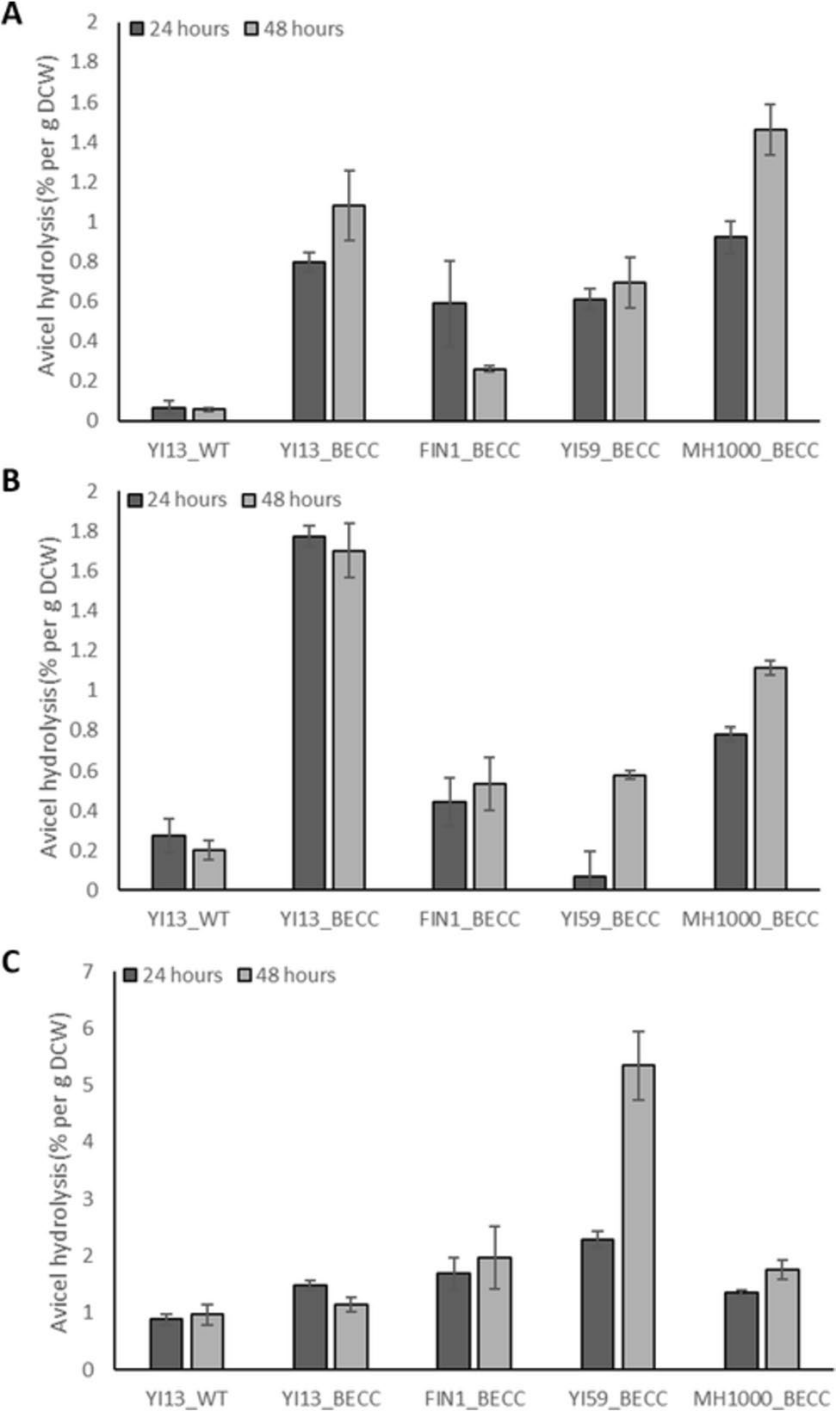
Avicel conversion efficacy of cellulases secreted by natural and industrial *Saccharomyces cerevisiae* isolates. Differences in conversion efficiencies achieved by the different strains was evaluated using supernatant from different cultivation conditions, namely, **A** optimal temperature 30 °C, **B** high temperature 37 °C, and **C** in the presence of 3 g/L acetic acid. Assay conditions were the same in all cases. Results represent means of triplicate assays and standard deviation is indicated with error bars

To evaluate the rudimentary cellulase system created for each of the strains, supernatant activity against microcrystalline cellulose (Avicel) was tested. Significant differences were observed between the reference strain and natural isolates, and between the cultivation conditions. As expected, the superior secretory capacity for *T.r.*EG2, *T.e.*CBH1, and *A.a.*BGL1 at optimal conditions and at higher temperature allowed for a high Avicel conversion efficiency by YI13_BECC at 48 h (1.08% and 1.70% per g DCW); however, somewhat surprisingly, the conversion efficiency was 1.35-fold lower than that of MH1000_BECC at optimal cultivation conditions (Fig. 3A). The better conversion efficiency obtained for MH1000_BECC may be due to strain background, as domestication events may have improved some phenotypic characteristics of the isolate (Mukherjee et al. 2014). In addition, the cellulases secreted by MH1000_BECC may have been present in a more optimal ratio to allow for a higher conversion efficiency; however, this cannot be confirmed as no gene copy number or transcriptomic analysis was performed and the amount of the CBH2 activity present could not be measured directly. Similarly, MH1000_BECC exhibited a higher efficacy than YI59_BECC and FIN1_BECC (1.93- and 2.09-fold, respectively) when cultivated at higher temperature (Fig. 2B), despite comparatively low CBH1 and BGL1 activity levels in this condition (Fig. 2C and E). The higher conversion efficiencies observed for this strain might suggest that superior production of the CBH2 enzyme along with the other cellulases was achieved in this strain background.

Another interesting result that arose from the Avicel conversion assays was the high conversion efficiencies observed for cellulases produced under acetic acid stress. As the wild-type negative control also had higher values in these conditions, we assumed that acetic acid carried over from the cultivation conditions affected the assay. Even so, the comparative levels of conversion were significantly higher in these conditions, with efficiencies that ranged between 1.14 and 5.34% per g DCW (Fig. 3C). YI59_BECC displayed the highest conversion efficiency (5.34% per g DCW), corresponding to a 3.04-fold higher efficiency than MH1000_BECC. Interestingly, FIN1_BECC displayed a similar conversion efficiency to that of MH1000_BECC, despite low enzyme activity levels achieved for the individual cellulases in this cultivation condition.

### Growth curve analysis

**Fig. 4:**
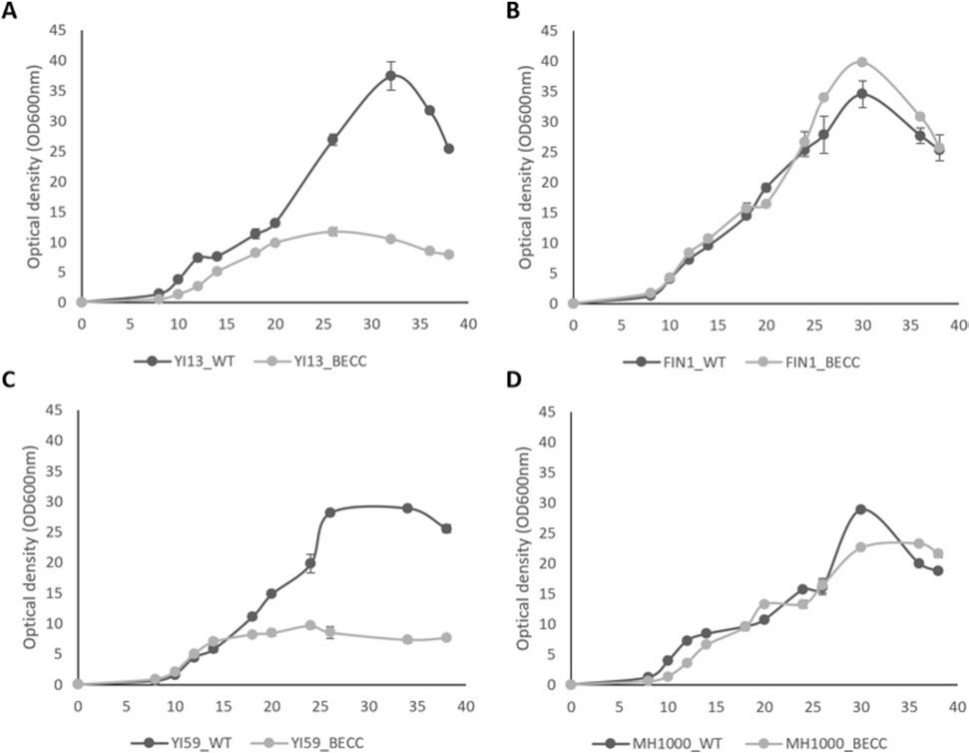
Growth curve analyses of wild-type and transformed natural and industrial *Saccharomyces cerevisiae* isolates*.* Isolates were cultivated in YP media supplemented with 2% (w/v) glucose and samples were taken in 2-h intervals for a 40-h growth period. Results represent means of triplicate cultivations and standard deviation is indicated with error bars

Growth profiles of transformed strains were evaluated to determine the effects of heterologous cellulase production on the normal metabolism of the cells. Growth profiles for YI13, FIN1, YI59, and MH1000 wild-type and transformed strains were evaluated in YPD liquid media at 30 °C over the course of 39 h (Fig. 4A–D). No significant variation could be observed between the wild-type and transformed isolates of FIN1 and MH1000. However, it was noted that the growth of YI13_BECC and YI59_BECC were significantly impaired compared to their untransformed counterparts. Growth for YI13_WT peaked at OD_600nm_ = 37.45, compared to the transformed YI13_BECC whose growth peaked at OD_600nm_ = 11.71 (Fig. 4A). Similarly, YI59_WT exhibited a peak in growth at OD_600nm_ = 28.89, while the growth peak for YI59_BECC was noted at OD_600nm_ = 9.68 (Fig. 4C). This was surprising, as high heterologous cellulase secretion levels was observed for these strains (Fig. 2) and suggested a metabolic burden because of constitutive production of the heterologous cellulases. As the YI13_BECC and YI59_BECC strains generally produced high enzyme activity levels, we assume they were exhibiting higher protein production levels at the expense of biomass production.

### Evaluation of strain robustness based on stress tolerance

**Fig. 5:**
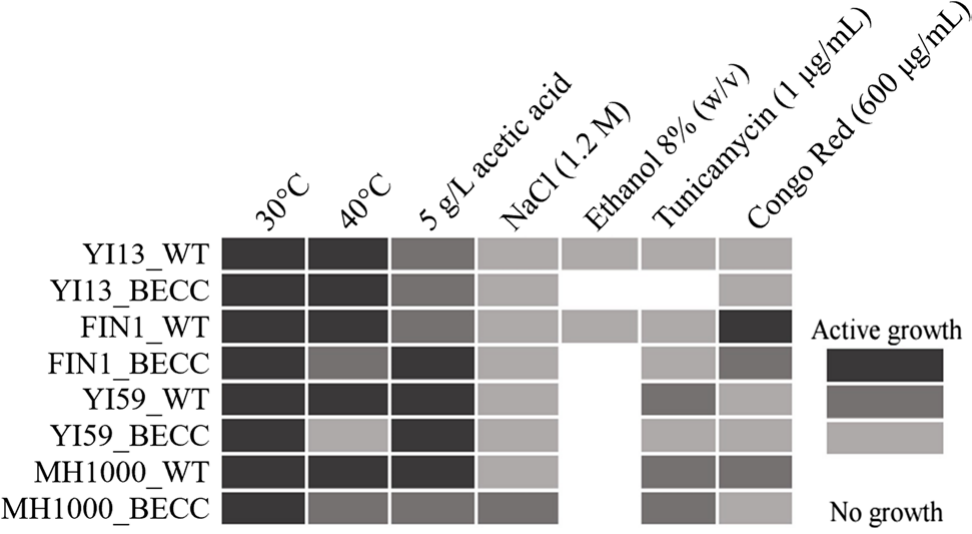
Heat map illustrating robustness of natural and industrial *Saccharomyces cerevisiae* strains against various second-generation (2G) bioethanol production stress and stresses related to heterologous protein production. Grey scale representing active to no growth was scored based on average dilutions over duplicate stress plates per strain. This is a graphical illustration of the plate images presented in Supplementary material Fig. S1

Developing bioethanol CBP processes not only requires the host strain to produce cellulases and high ethanol yields and titres but also demands tolerance to the harsh conditions inherent to this fermentation setting (Deparis et al. 2017). Therefore, strain tolerance capabilities to relevant stress conditions were evaluated to determine how heterologous cellulase production might have affected normal cell metabolism. In Fig. 5, the phenotypic diversity among untransformed and transformed natural isolates and the reference strain is illustrated with a grey scale, from most active growth to no growth at a given stress condition as indicated (additional data in Supplementary material Fig. S1). As illustrated, significant differences were observed between untransformed and transformed natural isolates, and in comparison, to the reference strain, MH1000. All the strains tested exhibited increased tolerance to high temperature and 5 g/L acetic acid, while growth in 1 M NaCl was less vigorous. In addition, no growth was observed for all the transformed strains when cultivated in the presence of 8% (w/v) ethanol, highlighting the increased sensitivity to high ethanol concentrations, which may have been caused by the metabolic burden exerted on strains by the heterologous cellulase production. Furthermore, increased sensitivity towards secretion- and cell wall stressors were observed for the transformed isolates compared to their untransformed counterparts, suggesting that heterologous protein production exerted an increased metabolic burden on the transformed strains. Surprisingly, YI13_WT and YI13_BECC exhibited increased sensitivity towards tunicamycin, despite the high cellulase activity levels achieved by the transformed isolate. Furthermore, this result contrasted with what was reported by Davison and co-workers (Davison et al. 2019b). The concentration of tunicamycin used in this study may have been too high for the strains. Moderate to high sensitivity was also observed for Congo Red, for both transformed and untransformed strains, which agreed with the high ethanol sensitivity observed. This suggested that despite the diverse strain backgrounds of the isolates screened, their membrane compositions did not allow for greater tolerance to high concentrations of ethanol.

### Fermentation of microcrystalline cellulose

**Fig. 6:**
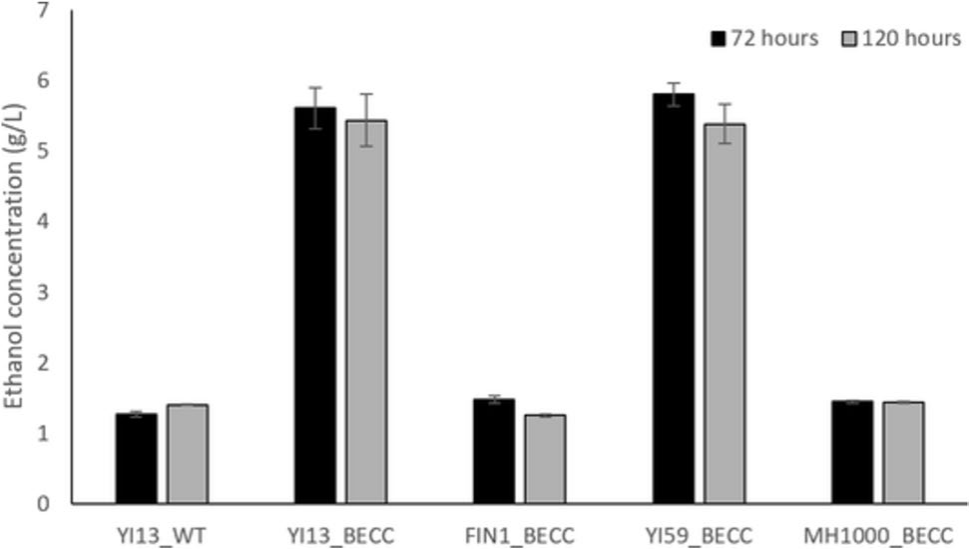
Ethanol concentrations (g/L) obtained by recombinant natural isolates of *S. cerevisiae* cultivated on YP-medium with 20 g/L Avicel as only carbohydrate. Results represent means of triplicate assays and standard deviation is indicated with error bars

Direct conversion of a crystalline cellulose substrate (Avicel) to ethanol in a CBP configuration without the addition of exogenous cellulases using our strains was subsequently attempted. Pre-cultured strains were inoculated on media with Avicel (20 g/L) as carbohydrate source in oxygen-deficient conditions for 120 h. Samples were periodically taken for HPLC analysis. Ethanol concentrations in the range of ~ 1–5.5 g/L was obtained, with YI13_BECC and YI59_BECC being the best ethanol producers, generating 35–40% of the theoretical maximum ethanol yield in this CBP configuration (Fig. 6). This agreed with enzyme activity evaluations illustrating that these two strains were the superior secretors for cellulases, also confirming that the secreted enzymes were active and functional to have achieved efficient Avicel hydrolysis.

Glycerol levels of less than 1 g/L were produced by YI13_BECC, YI59_BECC, and MH1000_BECC, which may have occurred because of increasing sugar or ethanol concentrations during the fermentation (Table 3). No detectable residual glucose was present at 120 h in any of the fermentations, which suggested all liberated glucose was utilised by the fermentative isolates. Residual cellobiose levels obtained were in the range of 0.82–1.47 g/L, suggesting EG2 and CBH1 were functional; however, the notable levels present at 120 h might also suggest that the BGL1 activity was not high enough; hence, EG2 and CBH1 may have been inhibited to some extent. Lastly, observed acetic acid levels in the range of 0.67–1.29 g/L may suggest production of the weak acid to neutralise the extracellular pH (Deparis et al. 2017).

**Table 3:** Metabolites produced by strains cultivated for 120 h on 20 g/L Avicel. Values are given in g/L, and represent means of triplicate cultivations with standard deviation as indicated

Yeast strains	Cellobiose	Glycerol	Acetic acid
YI13_WT	0.852 ± 0.003	0.621 ± 0.016	0.667 ± 0.048
YI13_BECC	1.302 ± 0.060	0.892 ± 0.057	1.013 ± 0.063
FIN1_BECC	0.818 ± 0.002	0.598 ± 0.004	0.701 ± 0.044
YI59_BECC	1.371 ± 0.060	0.730 ± 0.079	1.291 ± 0.032
MH1000_BECC	1.470 ± 0.027	0.787 ± 0.005	0.725 ± 0.015

## Discussion

Overexpression of heterologous proteins may result in a severe metabolic burden on cellular hosts, as energy and resources are redirected from normal metabolism to heterologous gene expression and enzyme production (Brandt et al. 2021). The extent of the metabolic burden in recombinant cellulase producing strains is dependent on various factors, such as gene copy number, source of the gene, expression strategy (i.e., secretion, cell-attachment, mini-cellulosomes, and/or combinations), oxygen availability, and the strain background. In addition, changes in the physical environment (temperature, pH, nutrients, solutes, and inhibitors) also contribute significantly to the viability of cells and the resulting heterologous protein production process (Brandt et al. 2021; Shen et al. 2020). We therefore investigated heterologous cellulase production in various strain backgrounds, previously shown to be robust to various CBP related stress factors, to determine how process relevant changes in the environment, which impact these strains differently, affected their ability to secrete recombinant enzymes and convert crystalline cellulose to ethanol.

### Effect of process conditions on heterologous enzyme secretion

Internal and external stresses were previously shown to impact secretion of recombinant proteins in natural isolates of *S. cerevisiae* (Davison et al. 2016, 2019a, b; Jansen et al. 2018). In our strain backgrounds, a range of secretory capacities was observed for individual cellulase enzymes secreted under various fermentation-associated process conditions (Fig. 2), with YI13_BECC and YI59_BECC exhibiting the highest secretory phenotypes, compared to the transformed reference commercial strain MH1000_BECC. Since these natural isolates were isolated from environments with harsh external conditions, natural adaptation had allowed them to diverge as organisms with greater genetic complexity, higher phenotypic variation, and higher tolerance to several environmental stresses (Davison et al. 2016; De Witt et al. 2019). One explanation for their increased heterologous protein production under stressful conditions is because of improved cell wall integrity, due to defects in key Golgi mannosyltransferase genes such as *OCH1* or *MNN9,* or disruption in genes encoding for cell wall mannoproteins and/or cell wall–related secretory proteins (Li et al. 2022). Adaptation events have allowed these isolates to enhance the expression of their cell wall proteins for added protection, i.e., having thicker cell walls to better protect internal organelles against external stresses. Furthermore, it was shown that a regulatory relationship likely exists between the cell wall integrity and the secretory pathways in yeasts (Arnthong et al. 2022; Li et al. 2019, 2022), suggesting that increased cell wall integrity could potentially limit the amount of stress induced to the secretory pathway. This suggests that these strains have developed a higher innate protein folding capacity which could alleviate stress that is induced to the endoplasmic reticulum (ER), hence reducing the impact exerted on the unfolded protein response (UPR) resulting in higher heterologous protein secretion levels (Davison et al. 2019b).

Several “-omics” studies have been performed on these natural strains and closely related counterparts (Davison et al. 2019b; De Witt et al. 2019; Davison 2019). Using this published data, we identified mechanisms that will allow for the improved secretion phenotypes observed in our study. Firstly, natural isolates carry allelic variants of genes related to glycosylation (e.g., *ALG1* and *ALG2*) that result in enhanced glycosylation efficiency, which promotes better heterologous protein secretion. Furthermore, variations in genes related to Golgi-associated retrograde transport, such as *VPS* genes, were identified that may influence protein secretion. For instance, disruption of *VPS10* was found to increase recombinant protein secretion levels. The heat shock response, a cellular defence mechanism against proteotoxic stress, enhances thermotolerance in certain natural isolates. Increased activity of the HSR-related transcription factor HSF1 led to higher expression of heat shock proteins in YI13-based strains, improving protein folding and secretion under stress conditions. This explains both the increased heat tolerance and increased heterologous secretion of YI13-based strains, as the HSR induces the production of chaperones that aid in correct protein folding. Finally, the *HAP1* gene, which regulates mitochondrial biogenesis and respiratory genes, was overexpressed in natural isolates. This activates genes involved in oxidative stress and respiration, reducing oxidative stress related to protein folding and enhancing heterologous protein secretion and tolerance to stresses such as weak acids.

Although some natural isolates hold promise for use as CBP organisms, they often lack some or most of the traits required for a superior industrial strain (de Witt et al. 2019), such as thermo- and osmo-tolerance, fermentation vigour, and tolerance to fermentation stresses. This was particularly evident in the fact that YI59_BECC yielded high heterologous protein secretion levels in the presence of acetic acid, however, relatively poor secretion titres when cultivated at high temperature (Fig. 2). Increased temperature sensitivity was also observed by Davison and co-workers (Davison et al. 2019a) and could be explained by inefficient induction of heat shock protein response (Jansen et al. 2018). As a result, cellular metabolism and growth was maintained at the expense of heterologous protein expression and secretion. Therefore, the range in secretory capacities observed between the recombinant host strains provides evidence that the diversity in genetic background plays a pivotal role in the expression and secretion levels of heterologous proteins. It follows that classical breeding of these strains may be useful to combine these positive traits in future.

### Cellulose conversion by heterologous cellulases

YI13_BECC and YI59_BECC displayed the highest overall cellulose conversion after cultivation at 37 °C and in the presence of acetic acid stress, respectively (Fig. 3B and C). These results directly correlate with individual cellulase activities, as both strains displayed relatively high cellulase activity levels under the same process conditions (Fig. 2). The increased conversion efficiencies could thus be explained by the high cellulase titres in these conditions, and an improved synergy among the secreted enzymes. In addition, the presence of the codon-optimised *Chrysosporium lucknowense* cellobiohydrolase (*C.l.*CBH2) may have contributed significantly to the high conversion efficiencies (Chetty et al. 2022). Since cellobiohydrolases are known to be the rate-limiting enzyme in cellulose hydrolysis (Ilmén et al. 2011), it became obvious from the improved conversion efficiencies that the CBH1 and CBH2 enzymes were both required to allow sufficient Avicel hydrolysis. Surprisingly, the conversion efficiency obtained for MH1000_BECC was also relatively high at optimal cultivation conditions, despite the strain having lower cellulase secretion capacities. This phenomenon can potentially be explained by the presence of the *C.l.*CBH2 enzyme working in synergy with the rest of the cellulase enzymes to have allowed for improved Avicel hydrolysis. However, we could not prove this due to the lack of a specific assay for the GH6 type of cellobiohydrolase when present among a mixture of cellulases.

### Effect of heterologous cellulases on strain growth

*S. cerevisiae* is well known for its use as recombinant cell factory to produce various proteins of interest; however, heterologous protein production often exerts cellular and metabolic stresses on the yeast cell (Davison et al. 2016; Deparis et al. 2017; Ilmén et al. 2011). These changes in the physiology of the yeast can result in obtaining low protein production levels, due to impaired growth and metabolism. Due to the variation in protein production levels achieved in this study, growth profiles of the strains were evaluated to determine the effects of heterologous cellulase production on growth.

No significant differences were observed between untransformed and transformed FIN1 and MH1000 strains during the 39-h growth period (Fig. 4B and D), which suggested that the constitutive expression of the heterologous cellulases did not have any detrimental effect on the growth and cellular metabolism of these strains. Conversely, growth curves showed that the superior cellulase secretors experienced significant decreases in their growth capability compared to their parental counterparts (Fig. 4A and C). In the first 8 h, similar growth patterns were observed for both untransformed and transformed strains; however, upon entering the exponential phase, the transformed strains grew at a slower rate compared to their untransformed counterparts. Metabolic burden exerted on these recombinant strains due to high heterologous protein production levels explains this phenomenon as the slower growth rate displayed by the transformed strains was likely a result of high heterologous cellulase production obtained at the expense of biomass production (Van Rensburg et al. 2012; Chetty et al. 2022), or delayed growth due to longer adaptation periods (lag phase) required by these strains (Deparis et al. 2017). Interestingly, the slower growth rate of YI13_BECC may have contributed to its reduced temperature sensitivity, as slow growing cells are less affected by heat stress (Gibney et al. 2013).

### Tolerance against fermentation-associated and secretion stresses

Stress tolerance exhibited by yeast strains is referred to as the collective response induced by the host organisms to adjust their normal metabolism to survive extreme stresses (Jansen et al. 2018). Adjusting metabolism involves optimising energy metabolism, amino acid biosynthesis, transportation of proteins and nutrients, and membrane integrity and fluidity. Exposure to high temperature was shown to induce the heat shock response, which induce the expression of heat shock proteins (HSPs) and accumulation of trehalose, to lend protection to cell membranes and proteins (Jansen et al. 2018). While having detrimental effects on cell membranes, high temperature also has a significant effect on regulation of enzymes, chaperones, and other proteins involved in protein processing (Chetty et al. 2022), which may affect protein production yields. All transformed strains experienced some sensitivity to high temperature (40 °C), except YI13_BECC which displayed active growth at both 30 °C and 40 °C (Fig. 5). This was corroborated by Jansen and co-workers (Jansen et al. 2018) who identified the maximum growth temperature of this strain to be 45 °C. Furthermore, the high heterologous protein secretion levels achieved by YI13_BECC at high temperature (Fig. 2) was likely enabled by activation of the heat shock response to relieve in ER stress in this strain background as demonstrated by Davison (2019), highlighting the close regulatory network between environmental stress and heterologous protein production (Hou et al. 2013). It is thus evident that despite the strain being subjected to temperature and heterologous protein production stress, YI13_BECC still managed to function, suggesting successful management of cellular metabolism and growth by the strain when exposed to these stressors.

Similarly, ethanol toxicity was shown to affect cell viability, metabolism, and growth, by targeting cellular membranes and protein and enzyme activity (Jansen et al. 2018). By altering membrane composition and fluidity, a reduction in proton-motive force occurs which compromises the uptake of nutrients into yeast cells (Deparis et al. 2017). This phenomenon was particularly evident when cultivating transformed and untransformed strains in the presence of 8% (w/v) ethanol (Fig. 5), where poor to no growth was observed after 48-h incubation. This result corroborates the hypothesis made by Jansen and co-workers (Jansen et al. 2018) who stated that exposure of yeast strains to an initial high ethanol concentration would lead to a significant loss of cell viability, compared to when cells are allowed to produce ethanol themselves to allow for gradual adaptation to the high ethanol content in their environment. However, in the case of YI13- and FIN1-based strains, an increase in sensitivity was observed between the untransformed and transformed strains, suggesting heterologous protein production may have contributed to the changed sensitivity in these strain backgrounds (Chetty et al. 2022).

High concentrations of weak acids, such as acetic acid, was shown to inhibit the growth and viability of *S. cerevisiae* (Fu et al. 2014; Deparis et al. 2017; Kawazoe et al. 2017), by acidifying the intracellular environment of cells (Zhang et al. 2019). This leads to accumulation of reactive oxygen species (ROS) and represses nutrient and energy utilisation in the cells. While active growth without any change in sensitivity were observed for YI59 untransformed and transformed strains, a notable increase in sensitivity was evident between the untransformed and transformed strains of FIN1 and MH1000 (Fig. 5). Diminished growth was observed as the cells redirected metabolism to maintaining normal cellular homeostasis and viability (Zhang et al. 2022).

While the environmental conditions in fermentations affect the growth and viability of yeast cells, heterologous protein production processes also affect cells negatively. We therefore tested the strains’ tolerances to secretion and cell wall stress, using chemical stressors to mimic secretory stress. Tunicamycin causes inhibition of *N*-linked glycosylation of nascent polypeptides in the ER, which allows accumulation of misfolded proteins and hence induces secretion stress (Chetty et al. 2022; Davison et al. 2019a). As a result, the chemical can be used to mimic or enhance heterologous protein induced stress in the ER. Poor to moderate growth was observed for untransformed and transformed strains (Fig. 5), with increased sensitivity displayed by the transformed strains. This was particularly evident for the superior cellulase secretors, YI13_BECC and YI59_BECC. This increased sensitivity can be explained by the metabolic burden imposed on the secretory pathway of the yeast cells. Similarly, the strains were also screened for the strength of their cell wall integrity, using Congo Red as chemical compound. As expected, all the tested strains displayed moderate to poor growth in the presence of 600 µg mL^−1^ Congo Red, which was evident of sensitivity to external stresses. This was also corroborated by Davison and co-workers (Davison et al. 2019a) who reported poor growth for the strains in question when cultivated in the range of 400–800 µg mL^−1^ Congo Red. This statement is supported by the observation of increased sensitivity to high concentrations of ethanol (8% w/v), salt (1.2 M NaCl), high temperature (40 °C), and acetic acid (5 g/L). It is worthy to note that although the superior cellulase secretors displayed varying tolerances to multiple inhibitors, it is supportive of the idea that no two strains have identical genetic and/or phenotypic backgrounds (Davison et al. 2016).

### Fermentation profiles

Direct conversion of crystalline cellulose (Avicel) to ethanol in a CBP configuration, without the addition of exogenous cellulases, was tested using our strains (Fig. 6). YI13_BECC and YI59_BECC yielded the highest ethanol titres of 4–4.5 g/L, compared to the reference industrial strain, MH1000_BECC, representing 35–40% of the theoretical maximum ethanol yield in this CBP configuration. This was expected, as these strains were identified as superior cellulase secretors for individual cellulase enzymes, as well as having high Avicel conversion efficiencies (Fig. 3). Our findings compare favourably with a study conducted by Liu and co-workers (Liu et al. 2017), who equiped an *S. cerevisiae* strain with a core set of cell-attached cellulases via a cocktail delta-integration method. Subsequent fermentation on crystalline cellulose (i.e., 10 g/L Avicel or 25 g/L pre-treated rice straw) yielded ethanol titres of 2.9 g/L and 0.8 g/L, respectively, without the addition of exogenous cellulase cocktails. In another study, ethanol yields of 0.93 g/L and 0.71 g/L was obtained when a recombinant *S. cerevisiae* strain co-expressing five cellulolytic genes was used in the fermentation of ionic liquid-pretreated bagasse and hardwood unbleached Kraft pulp (LUKP) (Amoah et al. 2017). Davison and co-workers (Davison et al. 2019b) reported construction of partially cellulolytic strains of *S. cerevisiae* that were grown on pre-treated corn residue substrates to evaluate the efficiency of the strains. Constructs co-producing EG2 and BGL1 in the same YI13 strain background used in our study converted 56.5% of the cellulose contained in the feedstock to fermentable glucose monomers. This resulted in an ethanol concentration of 4.02 g/L, without added exogenous cellulase cocktails. By combining individual cellulases required for efficient hydrolysis, an increase in synergy between cellulases could be achieved, resulting in higher ethanol yields. We can thus infer that, although successful construction of recombinant cellulolytic hosts has been achieved, low cellulase secretion titres still limit ethanol yields obtained. In addition, the harsh conditions presented in the fermentation with pre-treated lignocellulosic feedstocks may indicate reduced robustness of the utilised strains. Based on these previous reports, our results showed improved ethanol yields from microcrystalline cellulose (Avicel), which may relate to the diverse genetic backgrounds of the natural isolates utilised. However, to realise the true potential of the utilised natural strains, further improvements to cellulase expression and increased robustness would pave the way to obtain improved ethanol yields and titres from a wider range of substrates.

Comparison of heterologous cellulase production under optimal and stressed conditions demonstrated the diversity in strain backgrounds, as the strains exhibited varying tolerances and secretory capacities in different cultivation conditions. Fermentation profiles of transformed natural and industrial strains demonstrated the potential use of YI13_BECC and YI59_BECC as CBP hosts for bioethanol production. We can surmise that integration of the core set of cellulases into these strain backgrounds, with the use of CRISPR/Cas9 tools, allowed for the construction of superior recombinant cellulolytic hosts. Compared to recombinant strains constructed via conventional transformation strategies, the CRISPR/Cas9-constructs demonstrated sufficient capacity to heterologously produce cellulases to liberate glucose for fermentation to ethanol, while tolerating the harsh environmental conditions posed in the fermentation setting. In addition, the marker-free nature of the CRISPR/Cas9 approach allows the potential for further genetic manipulation to improve product yield, product range, and strain robustness. Hence, both the YI13_BECC and YI59_BECC strains can undergo additional manipulation and are suitable for classical breeding experiments. This insinuates the possibility of combining their positive traits, such as thermal tolerance, acid tolerance, and high protein secretion capacity, in the resulting offspring strains. Furthermore, it offers the potential for achieving improved ratios of the various cellulase encoding genes in the progeny strains. Harnessing the genetic diversity present in natural isolate strains holds the potential to develop more robust and efficient yeast strains for industrial processes, contributing to sustainable and economically viable bioproduction systems.

### Supplementary Information

Below is the link to the electronic supplementary material.

Supplementary file1 (PDF 316 KB)

## Data Availability

All data generated or analysed during this study are included in this published article and its supplementary information files.
