# Supplementary material for: Engineering natural isolates of Saccharomyces cerevisiae for consolidated bioprocessing of cellulosic feedstocks
Source: Appl Microbiol Biotechnol. 2023 Sep 9;107(22):7013–28. doi: 10.1007/s00253-023-12729-4 (PMC10589140; doi:10.1007/s00253-023-12729-4)
Supplement: Supplementary file 1 — Supplementary file1 (PDF 316 KB) [file 253_2023_12729_MOESM1_ESM.pdf]

Supplementary material for:

Engineering natural isolates of *Saccharomyces cerevisiae* for consolidated bioprocessing of cellulosic feedstocks

Letitia Minnaar[1] and Riaan den Haan[1*]

[1]Department of Biotechnology, University of the Western Cape, Bellville, South Africa

[*]Corresponding author: Tel no.: +27 21 959-2199 Email address: rdenhaan@uwc.ac.za

Letitia Minnaar ORCID: 0000-0001-5237-3528

Riaan den Haan ORCID: 0000-0001-6983-6226

Table S1: Primer sequences

Primer name	Sequence (5'- 3')	Application
Ch10.ENO1$_P$-L	GCAGTTATCTCTGTGTCCAGATCCCTT	Amplify homology repair template DNA ($ENO1_P$-*T.r.EGII*-$ENO1_T$) with Chromosome 10 target homology
Ch10.ENO1$_T$-R	CTACAGTAATTGTGCGGTGCAGGGAGG	
DELTA-ENO1-L	CTTAAGATGCTCTTCTTATTCTATTAAAAATAGAAAAT GACTTCTAGGCGGGTTATCTACTG	Amplify homology repair template DNA ($ENO1_P$-*T.e.CBHI*-$ENO1_T$) with Delta (Δ) target homology
DELTA-ENO1-R	GTTTGTTTGCGAAACCCTATGCTCTGTTGTTCGGATTTG ACGTCGAACAACGTTCTATTAGG	
DELTA_PGK1p-L	CTTAAGATGCTCTTCTTATTCTATTAAAAATAGAAAAT GATCCCTCCTTCTTGAATTG	Amplify homology repair template DNA ($PGK1_P$-*C.l.CBHII*-$PGK1_T$) with Delta (Δ) target homology
DELTA_PGK1p-R	GTTTGTTTGCGAAACCCTATGCTCTGTTGTTCGGATTTG AAACGCAGAATTTTCGAG	
Chr11 int-ENO-L	TGTAAAACAGGTATTGGCTGCTTCATAGTACACCCAAT TGCTTCTAGGCGGGTTATCTACTG	Amplify homology repair template DNA ($ENO1_P$-*S.f.BGLI*-$ENO1_T$) with Chromosome 11 target homology
Chr11 int-ENO-R	GCAACTCTGAAATGTCAAAACGGTCGTGTATAAATAAA TGCCGTCGAACAACGTTCTATTAGG	
Chr11 int-PGK-L	TGTAAAACAGGTATTGGCTGCTTCATAGTACACCCAAT TGTCCCTCCTTCTTGAATTG	Amplify homology repair template DNA ($PGK1_P$-*S.f.BGLI*-$PGK1_T$) with Chromosome 11 target homology
Chr11 int-PGK-R	GCAACTCTGAAATGTCAAAACGGTCGTGTATAAATAAA TGAACGCAGAATTTTCGAG	
Ch11_SEDp-L	TGTAAAACAGGTATTGGCTGCTTCATAGTACACCCAAT TGATTGGATATAGAAAATTAACGTAAGGCAGTATC	Amplify homology repair template DNA ($SED1_P$-*A.a.BGLI*-$DIT1_T$) with Chromosome 11 target homology
CH11_DITt-R	GCAACTCTGAAATGTCAAAACGGTCGTGTATAAATAAA TGTTACTCCGCAACGCTTTTCTG	
**Confirmation of gene integrations**		
EGR-Rev	ATCTGGATTAGTAACTTGAGACAAAGCAG	Confirm *T.r.EGII* integration in transformed strains
ENO1-L	GTAACATCTCTCTTGTAATCCCTATTCCTTCTAGC	
CBHIR-Rev	TGTTGAGAGAAGTCGTCGGTGTCAC	Confirm *T.e.CBHI* integration in transformed strains
ENO1-L	GTAACATCTCTCTTGTAATCCCTATTCCTTCTAGC	
CLCBHII-L	AGTCTTAATTAAACAATGGCCAAGAAGTTGTTC	Confirm *C.l.CBHII* integration in transformed strains
CLCBHII-R	AGTCGGCGCGCCTTAGAATGGTG	
BGLR-Rev	GGTTCATCATGTAAGAGTTTTCGC	Confirm $ENO1_P$-*S.f.BGLI*-$ENO1_T$ integration in transformed strains
ENO1-L	GTAACATCTCTCTTGTAATCCCTATTCCTTCTAGC	
BGLR-Rev	GGTTCATCATGTAAGAGTTTTCGC	Confirm $PGK1_P$-*S.f.BGLI*-$PGK1_T$ integration in transformed strains
PGK-L	CTAATTCGTAGTTTTTCAAGTTCTTAGATGC	
Ch11_SEDp-L	TGTAAAACAGGTATTGGCTGCTTCATAGTACACCCAAT TGATTGGATATAGAAAATTAACGTAAGGCAGTATC	Confirm *A.a.BGLI* integration in transformed strains
CH11_DITt-R	GCAACTCTGAAATGTCAAAACGGTCGTGTATAAATAAA TGTTACTCCGCAACGCTTTTCTG	
**Confirmation of chromosomal integrations at correct loci**		
Ch.10 Check-L	GCAGTTATCTCTGTGTCCAGATCC	Confirm *T.r.EGII* integration at Chromosome 10 target site/loci
T.r.EGII-R	GTACGGCGCGCCTTATAACTTTCTAGCCAAACATG AAGAAAG	
DeltaCheck-L	CTGTTGGAATAAAAATCCACTATCGTC	Confirm *T.e.CBHI* integration at delta (Δ) target sites/loci
ENO1-R	GCAACCCTATATAGAATCATAAAACATTCGTGA	
DeltaCheck-L	CTGTTGGAATAAAAATCCACTATCGTC	Confirm *C.l.CBHII* integration at delta (Δ) target sites/loci
CLCBHII-R	AGTCTTAATTAAACAATGGCCAAGAAGTTGTTC	
Ch.11Check-L	GCCTTCGATTTGACACATCTCTAAGC	Confirm *S.f.BGLI* integration at Chromosome 11 target site/loci
ENO1-R	GCAACCCTATATAGAATCATAAAACATTCGTGA	
Ch.11Check-L	GCCTTCGATTTGACACATCTCTAAGC	Confirm *S.f.BGLI* integration at Chromosome 11 target site/loci
PGK-R	ACTATTATTTTAGCGTAAAGGATGGGG	
Ch.11Check-L	GCCTTCGATTTGACACATCTCTAAGC	Confirm *A.a.BGLI* integration at Chromosome 11 target site/loci
CH11_DITt-R	GCAACTCTGAAATGTCAAAACGGTCGTGTATAAATAAA TGTTACTCCGCAACGCTTTTCTG	

Table S2: Yeast strains constructed in this study and their respective culture collection numbers at the publicly accessible Biobanks South Africa Yeast Culture Collection, Department of Microbiology and Biochemistry, University of the Free State. For more details, refer to table 1 in the main text.

S/N	Accession Form Nr	UFS Collection Nr	Other Nr (Strain Nr)
S. cerevisiae MH1000 transformed with *T.r.EG2; A.a.BGL1; C.l.CBH2* & *T.e.CBH1*	3070	UOFS Y-3424	MH1000_BECC
S. cerevisiae YI13 transformed with *T.r.EG2; A.a.BGL1; C.l.CBH2* & *T.e.CBH1*	3071	UOFS Y-3425	YI13_ BECC
S. cerevisiae FIN1 transformed with *T.r.EG2; A.a.BGL1; C.l.CBH2* & *T.e.CBH1*	3073	UOFS Y-3427	FIN1_ BECC
S. cerevisiae YI59 transformed with *T.r.EG2; A.a.BGL1; C.l.CBH2* & *T.e.CBH1*	3072	UOFS Y-3426	YI59_ BECC

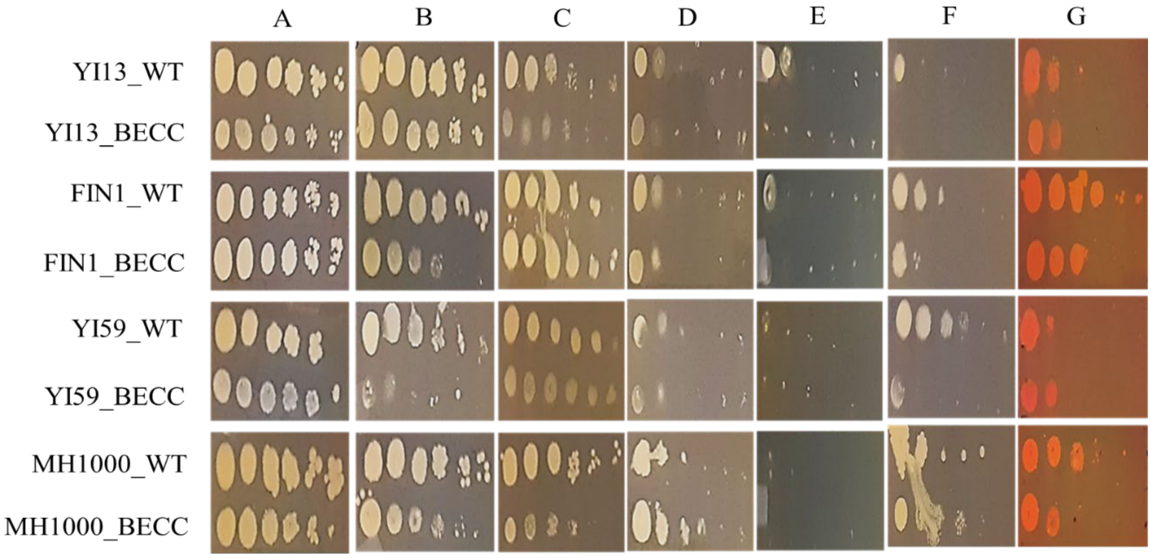

Figure S1: Tolerance of isolates against common bioethanol process-related stresses. Isolates were cultivated on YPD media supplemented with an appropriate inhibitor for 48 hours at 30°C, unless otherwise noted. Incubation at (A) 30°C; (B) 40°C; (C) evaluation of tolerance towards 5 g/L acetic acid; (D) evaluation of osmo-tolerance by cultivation on 1.2 M NaCl; and (E) evaluation of strain tolerance to the presence of an 8% (w/v) ethanol, (F) Tunicamycin (1 µg/mL) and (G) Congo Red (600 µg/mL). Dilutions are read from left to right, with a starting optical density of OD600nm = 1, followed by 10-fold dilutions thereafter.
